# Nested structure is dependent on visitor sex in the flower‒visitor networks in Kyoto, Japan

**DOI:** 10.1002/ece3.8743

**Published:** 2022-03-22

**Authors:** Shigeki Kishi

**Affiliations:** ^1^ Research Center for Agricultural Information Technology National Agriculture and Food Research Organization Minato Tokyo Japan

**Keywords:** ecological community, niche overlap, partner diversity, pollinator, sex

## Abstract

The characteristics of flower‒visitor networks, comprised of multiple species interacting with each other, predict ecological and evolutionary processes. Intraspecific and interspecific variations in interaction patterns should affect network structures. Because female and male visitors usually differ in flower‐visiting patterns due to mating strategy, visitor sex should affect nestedness, in which specialist species interact with a subset of species that interact with generalist species. I hypothesized that a network of male visitors and flowering plants would be more nested than a female network because males are less picky about which flowers they visit. To examine the effect of visitor sex on nestedness, I used museum specimens of insects and built 11 flower–visitor species networks, each composed of female and male subnetworks, and compared the strength of nestedness and related network metrics between the subnetworks. I found that male subnetworks were significantly more nested than female ones, and species networks were less nested than male or female subnetworks. The result may be attributable to the by‐chance selection of flowers by males. Because a nested structure is predicted to promote community stability in mutualistic flower–visitor networks, the greater nestedness of male subnetworks may suggest a positive effect of male visitors on pollination community stability.

## Graphical abstract

Male visitor's subnetworks were more nested than female visitor's subnetworks, while those two subnetworks cooperatively weakened nested structure of the original species networks.

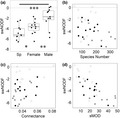

## 1 INTRODUCTION

An ecological community network is usually expressed as an assembly of species nodes and their interaction links. By measuring network structures, we can enhance our understanding of ecological and evolutionary processes in an ecological community (Montoya et al., 2006; Vázquez et al., 2009). Ecological networks share various characteristic structures (Bascompte & Jordano, 2007, 2013). Among these, nestedness is a representative structure commonly observed in flower–visitor networks (Bascompte et al., 2003), ectoparasite–vertebrate host networks (Graham et al., 2009), and resource–consumer networks (Kondoh et al., 2010). A nested structure emerges when specialist species tend to interact with a subset of the species that interact with generalist species (Ulrich & Gotelli, 2007; Wright & Reeves, 1992). Thus, a more‐nested network has relatively fewer specialist–specialist interactions than a less‐nested network. In mutualistic networks such as flower–visitor networks, a nested structure has been shown theoretically to promote network resilience, that is, a quick return to equilibrium after a disturbance, because redundant interactions in a nested structure prevent chained extinctions in such networks (Thébault & Fontaine, 2010). In antagonistic networks such as host–parasite networks and resource–consumer networks, however, a nested structure decreases network persistence, another aspect of stability, because it spreads adverse effects over such networks (Saavedra & Stouffer, 2013; Thébault & Fontaine, 2010).

The strength of a nested structure is dependent on interspecific and intraspecific variations of component species (Bascompte & Jordano, 2013; Tur et al., 2014). In particular, because considerable intraspecific variation has often been observed in field studies (Bolnick et al., 2011), intraspecific link variation would be necessary to be considered. Those can affect network structures and the ecological and evolutionary dynamics (Bascompte & Jordano, 2013; Bolnick et al., 2011). For example, when mutualistic and antagonistic interactions are mixed in a network, the effects of nestedness on the dynamics, resilience, and persistence are weakened (Sauve et al., 2014). Yet, there are few studies that surveyed the effects of intraspecific variation on network architectures, though increasing empirical studies have shown significant effects of intraspecific variation on the community dynamics (reviewed by Bolnick et al., 2011).

**FIGURE 1:**
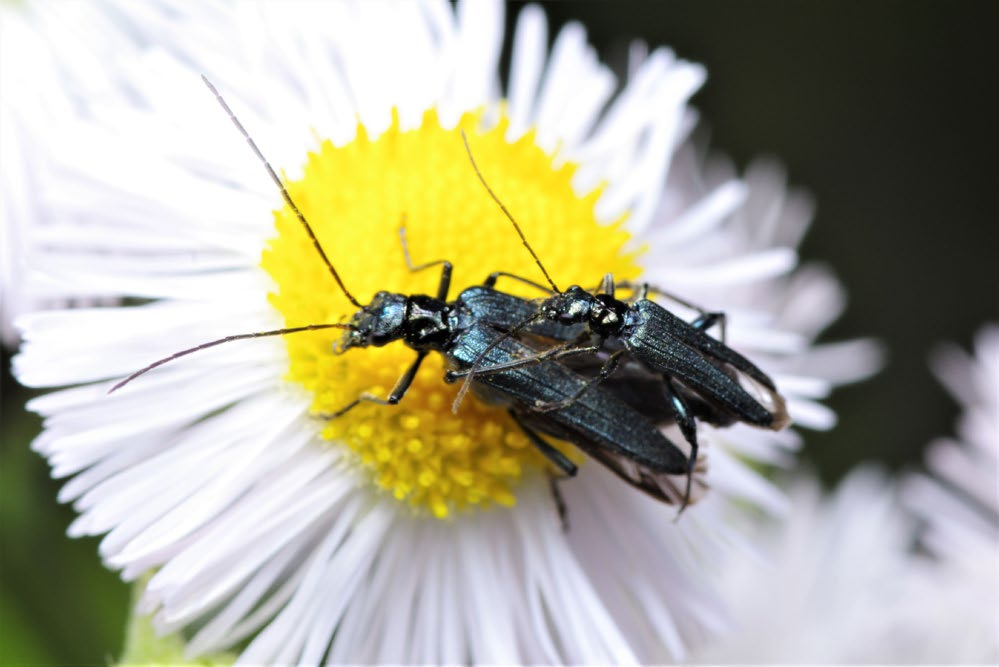
A pair of an Oedemerid beetle, *Oedemera lucidicollis*, on a flower of a Philadelphia fleabane, *Erigeron philadelphicus*. Female and male visitors would differently affect nestedness and other architectures of those flower‐visitor networks

Sex is one of the most distinctive factors underlying intraspecific behavioral variation (Fuster & Traveset, 2020). Some theoretical studies have reported that the population dynamics differ greatly between a sex‐separated network and an asexual network (Boukal et al., 2008; Doebeli & Koella, 1994). In a flower–visitor network, male and female visitors often differ in their flower‐visiting patterns (Ne’eman et al., 2006; Roswell et al., 2019; Smith et al., 2019) because males often prioritize mate‐searching whereas females prioritize searching for food resources to support reproduction (Goulson, 1999; Kevan & Baker, 1983; Figure 1). For example, males of many bees, beetles, butterflies, and hoverflies may exhibit territorial behavior when visiting flowers (Baker, 1983). Males of a large carpenter bee, *Xylocopa* (*Koptortosoma*) *ogasawarensis*, exhibit territorial behavior and repeatedly visit inflorescences of *Scaevola sericea* in their territories, both to feed and to search for females, which visit not only these inflorescences but also other various flower species to collect pollen and nectar (Sugiura, 2008). Others that do not exhibit territorial behavior may haphazardly search for female partners rather than flower rewards. Flower visitations by males are then likely to be less related to the quality and quantity of the flower rewards and rather more probabilistic, that is, more related to the encounter probability than visitations by females (Ne’eman et al., 2006; Roswell et al., 2019; Smith et al., 2019). Since interspecific interactions in response to the relative species abundance make the species network highly nested (Krishna et al., 2008), the flower‐visitation pattern of males may also make the network more nested. The sexual behavioral differences may also affect their likelihood of visiting flowers in the network. Males of a small flower beetle, *Nipponovalgus angusticollis*, visit many different flower species, whereas females spend their whole life in decaying wood and do not visit flowers at all (Kobayashi, 1983).

Recently, we showed that modularity, another representative network metric to evaluate the relative number of clusters (modules), differed between the sexes, such that male subnetworks were less modular than female subnetworks or the original species networks (Kishi & Kakutani, 2020). However, how modularity affects nestedness is dependent on other network attributes. Fortuna et al. (2010) showed that when connectance, the ratio of observed links to all possible links, was low, the correlation between nestedness and modularity was positive. It suggests that the strength of nestedness of male subnetworks may be higher than that of female subnetworks in the dataset of Kishi and Kakutani (2020), in which the average connectance was low. However, local interspecific and intraspecific interactions affect the correlation between the two (Valverde et al., 2020). Then, comparing the nestedness strength between female and male visitor subnetworks would give us worthful results to understand mutualistic flower‐visitor networks.

I hypothesized that the strength of the nestedness of male‐visitor subnetworks should be greater than that of female‐visitor subnetworks. To examine this hypothesis, I used records of flower‐visiting insect specimens in the Kyoto University Museum collections. The data was divided into 11 datasets according to the specimen collection site and year. At first, I compared the proportions of visitor species in each dataset consisting of all female individuals, male individuals, and both sexes. Then, for each dataset, I constructed a species–species network and divided it into a male subnetwork and a female subnetwork. Then, I compared the degree of nestedness and related metrics among them.

## 2 MATERIAL AND METHODS

### 2.1 Dataset

To compare network structures between male and female subnetworks, I used the identical flower–visitor data as Kishi and Kakutani (2020) (opened at the Dryad data repository). These data are based on specimens of flower‐visiting insects collected by T. Inoue, T. Kakutani, M. Kato, and their students at three sites, Ashu (35°18′34″N, 135°43′01″E), Kibune (35°07′19″N, 135°45′47″E), and the Yoshida campus of Kyoto University (35°01′34″N, 135°46′51″E), all of which are located in Kyoto prefecture (Inoue et al., 1990; Kakutani et al., 1990; Kato et al., 1990). The distance between the northernmost (Ashu) and the southernmost (Kyoto University) site is approximately 32 km, and Kibune is at an intermediate location. At each site, flower‐visiting insects were collected using insect nets along a census route one to three times each month from April to early November of each collection year. When a visitor individual was collected, the flower species that it was visiting at the time of collection was also recorded. Data of these specimens have previously been used for network studies (e.g. Bascompte et al., 2003; Dormann et al., 2009; Olesen et al., 2007), but without identifying their sex. Although according to these published reports, a total of 9171 individual flower visitors were collected at these three sites, just 6031 specimens are registered in the Kyoto University Museum's database, probably because some samples were lost or became deteriorated after their collection. Most of the specimens are identified to the species level. For further information, readers should refer to published reports (Inoue et al., 1990; Kakutani, 1994; Kakutani et al., 1990; Kato et al., 1990).

During August–December 2014, I checked the present status of each insect specimen and identified its sex if it had not been identified before. To make up datasets, I discarded data of specimens lacking record labels, and registered specimens that could not be found in the museum cases and specimens that were not collected on plants. I also discarded the data of specimens whose sex could not be identified. I retained the data of specimens classified only at the genus level (i.e., “sp.”). The final dataset comprised the data of 5212 individual insect specimens with complete records (species name, sex, collection date and site, and plant species name; Table 1). These 5212 insects were classified into 1099 species and were captured on 247 different plant species; 3256 individuals (710 species) were females, and 1956 individuals were males (618 species). Using these data, I constructed 11 species‐based networks (species networks) (one for each collection year and site) and then broke each of them down into a male subnetwork and a female subnetwork (Table 1; see also Tables S1 and S2, and Supplementary Tables in Kishi and Kakutani (2020)).

**TABLE 1:** Numbers of female (F) and male (M) flower visitors, collected at each of three research sites in each of four years, used for the network analyses. No field research was carried out at the Kyoto University site in 1984

Year	Research site						Total
	Kibune		Ashu		Kyoto University		
	F	M	F	M	F	M	
1984	395	292	248	210	–	–	1145
1985	207	155	124	44	253	131	914
1986	364	252	356	175	287	181	1615
1987	740	358	145	72	137	86	1538
Total	1706	1057	873	501	677	398	5212
	2763		1374		1075		

### 2.2 Network indices

To calculate network indices and analyze them, I used the open statistical software R version 3.6.2 (R development core team, 2020). The network indices were calculated by using the “bipartite” package version 2.15 (Olesen et al., 2007), and the GLMM analyses were carried out with the “lme4” package (Bates et al., 2015).

**FIGURE 2:**
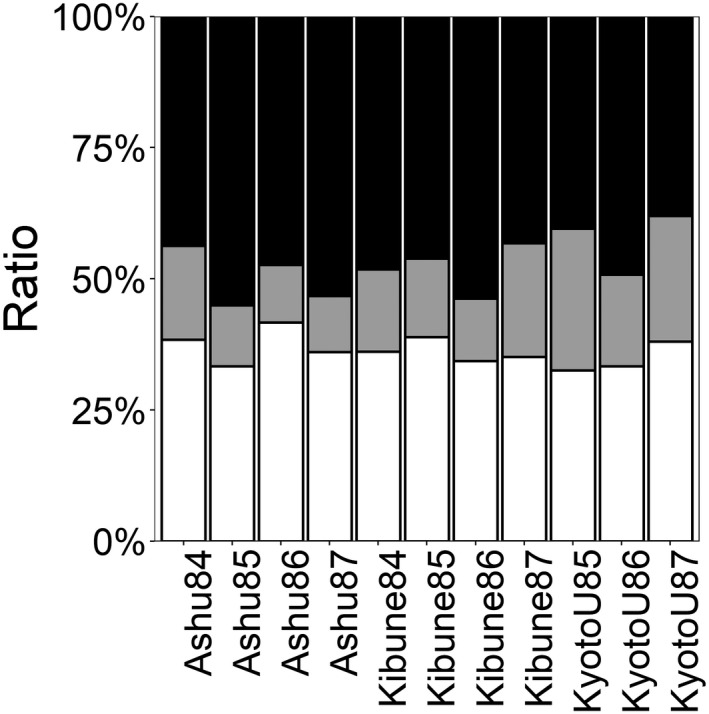
The proportions of visitor species consisting of all males (white), all females (black), and both sexes (gray) in each of the 11 flower–visitor networks (indicated by the collection site and year)

First, I counted the number of visitor species consisting of all females, all males, or both sexes (Figure 2) in each of the 11 species networks and determined the proportion (%) of species in each sex composition category in each network. I then used a generalized linear mixed model (GLMM) to compare the proportions of the three species sex composition categories (all females, all males, or both sexes) in the 11 networks. In this analysis, the explanatory variable was the species sex category (all females, all males, or both sexes), the response variable was the number of species in the category, network origin (the place and year of collection) was a random effect, and the offset was the total number of visitor species in the network. If a significant effect of the species sex category was detected, I then carried out multiple pairwise comparisons of the species sex categories by applying the Bonferroni correction (threshold value, *p* = .05/3 = .0167).

Next, for the species networks (including both males and females) and the male and female subnetworks, I calculated the weighted NODF (nestedness metric based on overlap and decreasing fill) value (Almeida‐Neto et al., 2008; Almeida‐Neto & Ulrich, 2011) as a measure of the degree of nestedness (Table 2). This widely used metric expresses the average degree of unilateral overlap between any pair of rows and that between any pair of columns based on frequency (weighted) data (Almeida‐Neto et al., 2008; Almeida‐Neto & Ulrich, 2011; Table 2). Because this value is known to be affected by the total number of species (i.e., network size), I standardized the weighted NODF values by using the *z* score (Almeida‐Neto et al., 2008; Bascompte & Jordano, 2013), which is the standardized distance from the mean of 1000 null networks, each generated from the original network using the r2d table algorithm (Dormann et al., 2009), which keeps the marginal sum of the matrix (i.e., the total interactions of each species) constant,

$$z=\frac{obs‐mean\left(\mathrm{nulls}\right)}{sd\left(\mathrm{nulls}\right)},$$

where *obs* is the observed value, *mean (nulls)* is the mean value of the null networks, and *sd (nulls)* is the standard deviation of null networks. Because a larger weighted NODF value (>0) indicates greater nestedness, the *z* score should be positive when the observed network is more nested than the null networks. I used a GLMM to examine whether the standardized weighted NODF (swNODF) value differed significantly among male, female, and species networks. In the GLMM, the response variable was the swNODF value, the explanatory variable was the network type (i.e., species network, female subnetwork, or male subnetwork), and network origin was a random effect. If a significant difference was detected, multiple pairwise comparisons of the three network types were carried out by applying the Bonferroni correction (threshold value, *p* = .05/3 = .0167).

**TABLE 2:** Network indices and the formulas used to compare male and female subnetworks

Index	Formula	Notes
Weighted Nestedness (weighted NODF) WNODF	$\mathrm{WNODFc}=100\sum_{i=1}^{n‐1}\sum_{j=i+1}^{n}\frac{k_{\mathrm{ij}}}{N_{j}}$ $\mathrm{WNODF}=\frac{2(\mathrm{WNODFc}+\mathrm{WNODFr})}{m\left(m‐1\right)+n\left(n‐1\right)}$	*i*, *j*: column (*c_i_*, *c_j_*) or row numbers (*r_i_*, *r_j_*) (*i* < *j*). *m*, *n*: total number of rows and columns, respectively. *k_ij_*: number of cells with lower values in *c_j_* than in *c_i_*. *N_j_*: total number of non‐empty cells in *c_j_*. WNODFc and WNODFr: mean nestedness values for all pairs of columns and for all pairs of rows, respectively.
Niche overlap 1 – *J*_chao_	$J_{\mathrm{Chao}}=\frac{{\hat{U}}_{A}{\hat{U}}_{B}}{{\hat{U}}_{A}+{\hat{U}}_{B}‐{\hat{U}}_{A}{\hat{U}}_{B}}$ ${\hat{U}}_{A}=\sum_{i=1}^{D_{\mathrm{AB}}}\frac{X_{\mathrm{iA}}}{n_{A}}+\frac{\left(n_{B}‐1\right)}{n_{B}}\frac{f_{+1}}{2f_{+2}}\sum_{i=1}^{D_{\mathrm{AB}}}\frac{X_{\mathrm{iA}}}{n_{A}}I\left(X_{\mathrm{iB}}=1\right)$	*n_A_*: number of species in the community (list of species abundances), *A*. *X_iA_*: number of individuals of species *i* in *A*. *D_AB_*: number of species shared between *A* and *B* communities. *f*_+1_, *f*_+2_: number of shared singleton and doubleton species. *I*: 1 if true, 0 if false.
Partner diversity $H_{0}^{\prime }$	$H_{j}^{\prime }=‐\sum_{i=1}^{n_{i}}p_{i}\ln\ln p_{i}$ $H_{0}^{\prime }=\frac{1}{\mathrm{SN}}\sum_{j=1}^{N}n_{j}H_{j}^{\prime }$	*p_i_*: proportion of interactions between the focal species and partner species *i*. *n_i_*: number of partners belonging to species *i*. $H_{j}^{\prime }$: Shannon's *H′* of species *j*. $H_{0}^{\prime }$: weighted mean of Shannon's *H′* at the visitor or plant level. *S*: total number of individuals at the visitor or plant level. *N*: total number of species at the visitor or plant level.

I also examined whether network size or connectance significantly affected swNODF because some studies have reported a positive relationship between nestedness and connectance, which is the ratio of the total number of observed links (cells) to the total number of cells in a bipartite matrix (i.e., all possible links; Song et al., 2017; Valdovinos et al., 2016). To test the effect of the total number of both plant species and visitor species (network size) on swNODF, I used a GLMM in which the response variable was the swNODF value, the explanatory variables were the total number of species and network type, and network origin was a random effect. To examine whether observed differences of swNODF among the three network types were related to connectance, I also used a GLMM to test the effect of connectance on swNODF. In this analysis, the response variable was the swNODF value, the explanatory variables were connectance and network type, and network origin was a random effect.

I used the Pearson product‐moment coefficient to test the correlation between the swNODF values and the standardized modularity (sMOD) values for each network type. The sMOD values were identical with the values used in Kishi and Kakutani (2020), to which more details may be referred.

To better understand and interpret the results of the nestedness comparisons, I calculated the degrees of niche overlap and partner diversity in visitors and plants of each network (Table 2). Niche overlap refers to the mean similarity in the interaction pattern between species subsets of visitors or plants (Dormann et al., 2009; Valdovinos et al., 2016). Because the species subsets with which visitors or plants interact would show greater similarity in a nested network, niche overlap is expected to increase with the degree of nestedness when visitors do not try to avoid competition (Valdovinos et al., 2016). The degree of visitor niche overlap was calculated as the average value of residuals calculated by subtracting the value of the Chao index from 1 (niche overlap = 1 – Chao index) for all possible pairs of visitor species (i.e., any two subsets of plant species each visited by the same single insect visitor species). The Chao index is a metric of the dissimilarity between any two subsets of species and ranges from 0 (perfect match) to 1 (no overlap) (Chao et al., 2005). Thus, in each network, the mean value of the residual (1 = Chao index) for insect visitor species (plant species) was used as the degree of visitor (plant) niche overlap. The degree of plant niche overlap was calculated in the same way for all possible pairs of plant species (i.e., any two subsets of insect species that each visited the same single plant species; Table 2).

Partner diversity was calculated by using the Shannon biodiversity index, *H’*, the value of which increases when a species interacts with a greater number of species more evenly (Blüthgen et al., 2006; Dormann, 2011). Both the plant diversity degree of the visitors (visitor partner diversity) and the visitor diversity degree of plants (plant partner diversity) were calculated. The degree of visitor (plant) partner diversity is the weighted mean value of *H’* for visitor (plant) species (Blüthgen et al., 2006). The degree of visitor partner diversity increases when many visitor species evenly visit more plants (visitor partners). All values of visitor niche overlap, plant niche overlap, visitor partner diversity, and plant partner diversity were also standardized by using the *z* score. Because the *z* value of visitor partner diversity and that of plant partner diversity were identical for each network when the 1000 null models were identical, I unified the two values into a single metric, partner diversity.

I used a GLMM to compare the standardized values of niche overlap and partner diversity among the three networks. In a GLMM, the response variable was either of the z scores of visitor niche overlap, plant niche overlap, or partner diversity, the explanatory variable was the network type, and network origin was a random effect. To examine whether each index of niche overlap and partner diversity was significantly correlated with swNODF, I used a GLMM in which the response variable was swNODF, the explanatory variables were a pair of network type and either visitor niche overlap, plant niche overlap, or partner diversity, and network origin was a random effect. When a significant difference was detected, multiple pairwise comparisons among the three networks were carried out by applying the Bonferroni correction (threshold value, *p* = .05/3 = .0167).

## 3 RESULTS

The GLMM result showed that the proportions of the species sex categories differed significantly (*F* = 32.09, *p* < .0001). In the 11 networks, 47.12 ± 5.53% (mean ± standard deviation) of visitor species consisted of only females, 36.18 ± 5.54% consisted of only males, and 16.7 ± 2.83% consisted of both sexes. In the multiple pairwise comparison tests, the proportion of visitor species consisting of only females was higher than the proportion composed of only males and the proportion including both sexes (female–male, *F* = 24.07, *p* < .001; female–both, *F* = 35.40, *p* < .001; male–both, *F* = 28.49, *p* < .001). The proportion consisting of only males was higher than the proportion consisting of both sexes, less than 20%.

**FIGURE 3:**
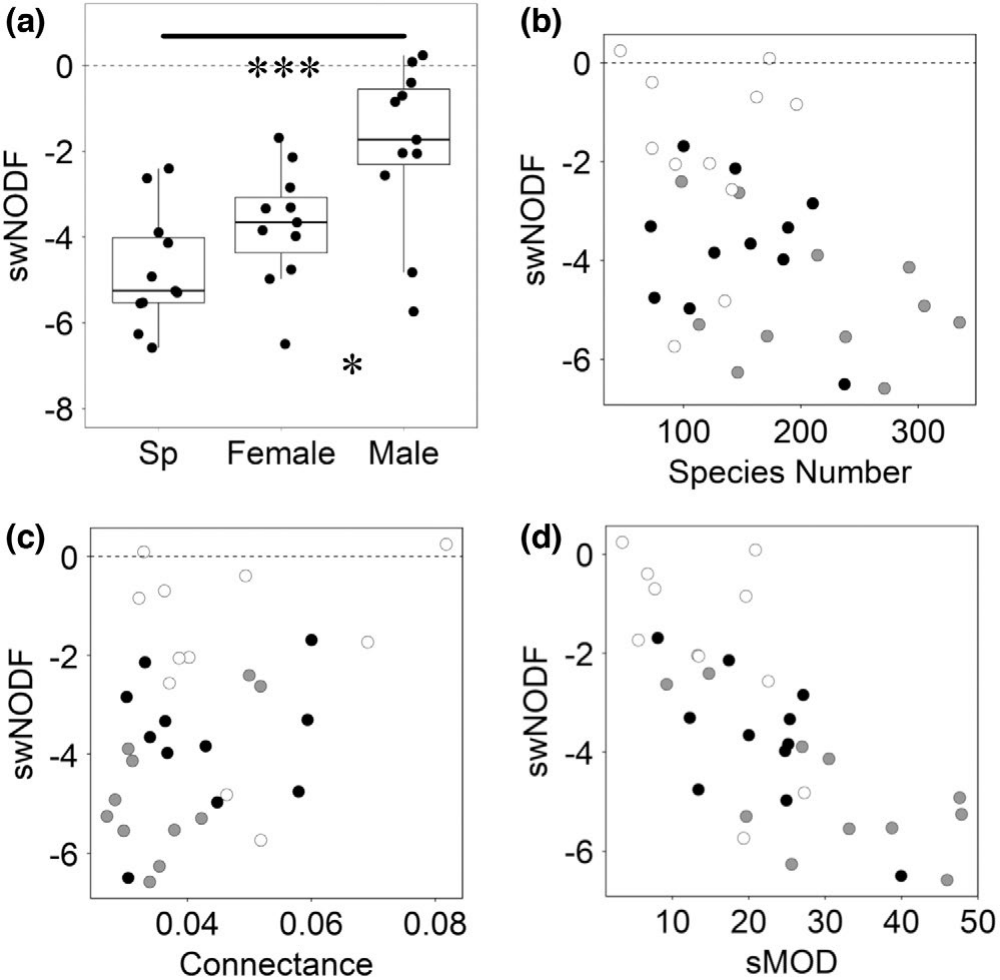
(a) Standardized values (*z* scores) of the weighted NODF (swNODF) of species networks (Sp, without regard to sex), female subnetworks (Female), and male subnetworks (Male). Black circles indicate the observed values. The bold horizontal line in each box indicates the median. The top and bottom lines of the box indicate the 75th and 25th percentiles, respectively. The whiskers indicate the maximum and minimum values. Significant differences are shown by asterisks (*p < .05; **p < .01; ***p < .001; GLMM). Scatter plots of swNODF versus (b) the total number of species in a network (Species Number) and (c) the connectance of species networks (gray circles), female subnetworks (black), and male subnetworks (white)

Most network *z* scores of weighted NODF (swNODF) values were negative (species, 11/11; female, 11/11; male, 9/11), indicating that those networks were less nested than weighted null models randomized by the r2d table algorithm (Figure 3a). The values of swNODF also differed significantly among the three network types (*F* = 13.31, *p* < .001, Figure 3a). The average swNODF value of male subnetworks (−1.87 ± 1.93) was within the 95% confidence interval (from −1.96 to 1.96), and was significantly higher than that of female subnetworks (−3.73 ± 1.35) and that of species networks (−4.77 ± 1.36; species–female, *F* = 6.71, *p* = .027 < .167; species–male, *F* = 32.03, *p* < .001; female–male, *F* = 6.88, *p* = .0162).

The GLMM analysis detected no significant effects of the number of species (*F* = 0.10, *p* = .75), network type (*F* = 0.35, *p* = .71), and the interaction effect (*F* = 0.34, *p* = .71) on swNODF (Figure 3b). Another one also detected no significant effects of connectance (*F* = 1.26, *p* = .28), network type (*F* = 3.18, *p* = .065), and the interaction effect (*F* = 1.01, *p* = .38; Figure 3c). Thus, swNODF values were not significantly affected by the number of species (network size) or by connectance. Meanwhile, the GLMM analysis detected significant correlation between sMOD and swNODF (*F* = 15.90, *p* < .01) but not effect of network type (*F* = 2.41, *p* = .12) and the interaction effect (*F* = 0.86, *p* = .44; Figure 3d).

**FIGURE 4:**
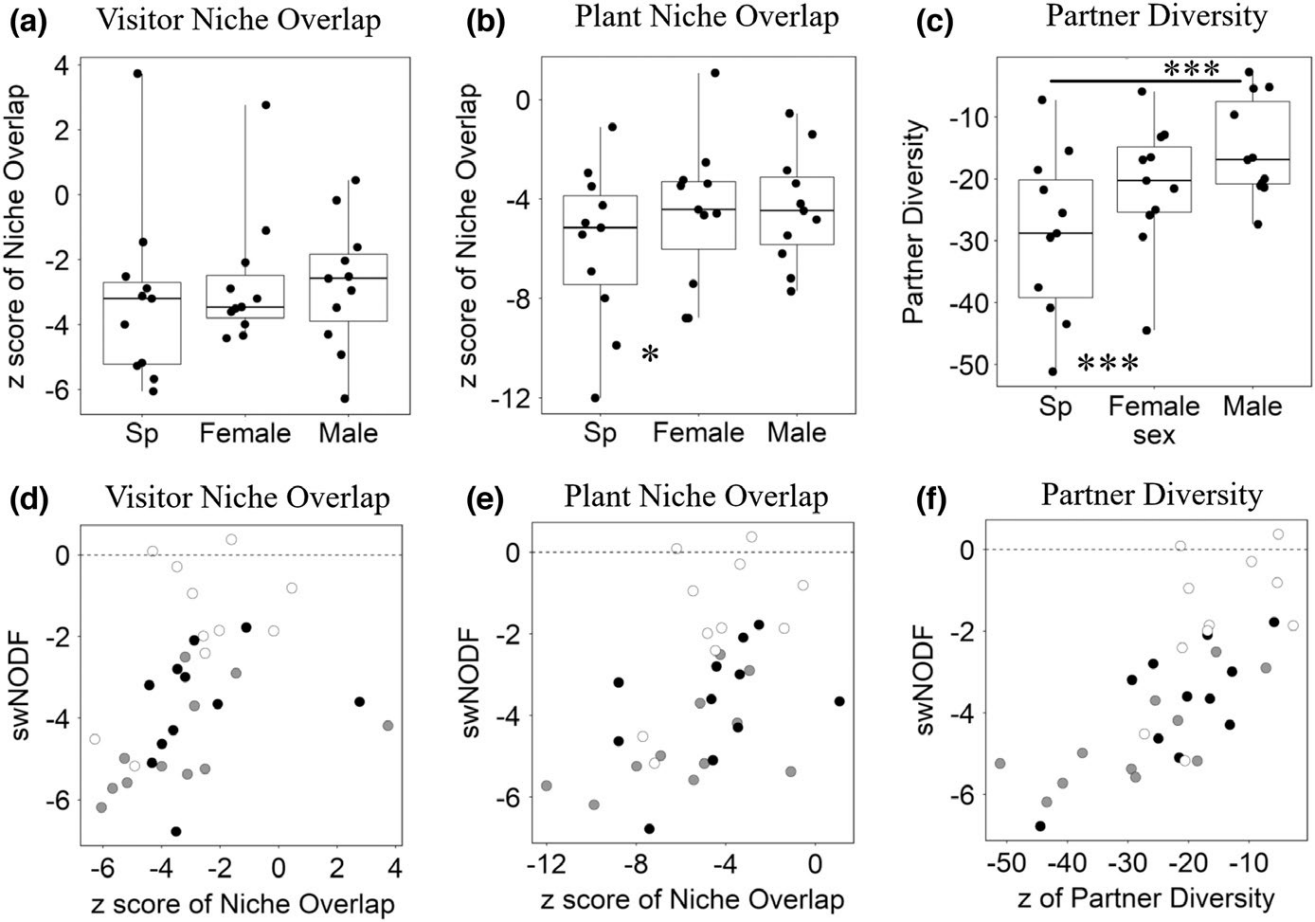
Standardized values of (a) visitor niche overlap, (b) plant niche overlap, and (c) partner diversity of species networks (Sp, without regard to sex), female subnetworks (Female), and male subnetworks (Male). Black circles indicate the observed values. The bold horizontal line in each box indicates the median. The top and bottom of the box indicate the 75th and 25th percentiles, respectively. The whiskers indicate the maximum and minimum values. Significant differences are shown by asterisks (*p < .05; ***p < .001; GLMM). Scatter plots of standardized weighted NODF (swNODF) versus (d) visitor niche overlap, (e) plant niche overlap, and (f) partner diversity for species networks (gray circles), female subnetworks (black), and male subnetworks (white)

The *z* scores of visitor niche overlap did not differ among the three networks (*F* = 1.11, *p* = .35, Figure 4a), whereas those of plant niche overlap differed significantly (*F* = 3.59, *p* = .047, Figure 4b). The *z* scores of plant niche overlap of female subnetworks were larger than those of species networks (species–female, *F* = 12.62, *p* = .0052; species–male, *F* = 5.75, *p* = .038; female–male, *F* = 0.056, *p* = .82). The *z* scores of partner diversity differed significantly among the three networks (*F* = 22.87, *p* < .001, Figure 4c). Those of male subnetworks and female subnetworks were larger than those of species networks, but the difference between those of female subnetworks and those of male ones was marginal (species–female, *F* = 26.85, *p* < .001; species–male, *F* = 39.46, *p* < .0001; female–male, *F* = 6.37, *p* = .030).

The GLMM analysis detected significant effects of the *z* scores of plant niche overlap (*F* = 8.39, *p* = .012) and the network type (*F* = 4.98, *p* = .018) on swNODF but not of the interaction effect (*F* = 0.64, *p* = .54; Figure 4d). Similarly, it detected significant effects of the *z* scores of visitor niche overlap (*F* = 4.67, *p* = .0498) and the network type (*F* = 7.42, *p* = .0043) on swNODF but not of the interaction effect (*F* = 0.38, *p* = .69; Figure 4e). Finally, it detected a significant effect of the z scores of partner diversity (*F* = 16.84, *p* = .0011) on swNODF, but not of the network type (*F* = 3.05, *p* = .071) and the interaction effect (*F* = 0.65, *p* = .53; Figure 4f).

## 4 DISCUSSION

The results showed that the structure of male subnetworks was more nested than that of female subnetworks, regardless of network size or connectance, neither of which was significantly correlated with swNODF. The average swNODF value of male subnetworks was in the 95% confidence interval, indicating that they were more similar to null networks in which the total number of individuals of each visitor species (column sums in a bipartite matrix) and the total number of visitor individuals (regardless of visitor species) to each plant species (row sums) were identical to the original networks. These results and the negative correlation between modularity and nestedness are consistent with Fortuna et al. (2010), as predicted. It suggests that male visitors tend to visit flowers according to encounter probability, or at least closer to statistical encounter probability, though it should be noticed that the species composition was considerably different between the two subnetworks.

The swNODF values of the species networks were not intermediate between the female and male subnetworks. Instead, they were the smallest among the three network types. This result may suggest that the two subnetworks jointly decreased the nestedness degree of the species networks. If preferences for flowers were similar among the two visitors, the nestedness degree of the species networks should be greater than the two subnetworks. Then, it may suggest that male visitors have different preferences from female visitors. Intraspecific link variation made by visitor sex may contribute to maintaining and increasing the community's biodiversity. However, the link variation may weaken the positive effects of nestedness on resilience and persistence, according to a theoretical study (Sauve et al., 2014).

Male and female subnetworks differed not only in their degree of nestedness but also in their species composition. On average, more than 45% of visitor species consisted of only males, and more than 35% consisted of only females, whereas less than 20% of species consisted of both sexes. The sex‐polarization of more than 80% of visitor species has several possible explanations. First, visitor species differ in their natural histories; only females or males visit flowers in some species. Second, the apparent sex polarization may also be merely stochastic because many species are represented in the database by only a few individuals. In particular, species represented by a single individual necessarily belong to only one sex. Third, in some solitary and social bees and wasps, the biased sex ratio of adults (Helms, 1994), together with stochasticity, would cause visitors of these species to exhibit sex polarization. Nevertheless, although sex polarization of visitor species may depend on the sampling effort, for some species, sex composition differences can be expected to occur in nature.

Standardized values of partner diversity of the species networks were smaller than those of both the female and male subnetworks. The Shannon diversity index *H*′, on which partner diversity is based, has two components: the skewness of the species abundance distribution (evenness) and the number of species (richness) (DeJong, 1975). Standardization by r2d null models should highlight the effect of skewed (specialized) interactions between visitors and plants more than the effect of the number of species with which each species interacts. Thus, this result indicates that more specialized interactions occurred in species networks than in the two subnetworks. The lower nestedness of the species networks would be attributable to the higher interaction diversity made by the two different subnetworks.

This study detected no differences in both visitor and plant niche overlaps between female and male subnetworks. However, plant niche overlap of species networks was smaller than that of female subnetworks. These results indicate that male visitors may be an agent to reduce plant niche overlap in the species networks and are consistent with the above results. Though there were no differences in both niche overlaps between the two subnetworks, positive correlations between either visitor and plant niche overlap and the nestedness degree were detected. These results suggest that visitor sex would not be a significant factor to form the correlation. It is consistent with a previous study that reported a positive correlation between niche overlap and the degree of nestedness in flower–visitor networks when adaptive foraging by visitors did not occur (Valdovinos et al., 2016). Thus, the correlations observed in this study may reflect interspecific interactions between visitors, such as competition, or seasonal and environmental discrepancies between visitors and plants. The negative values of standardized niche overlap in most networks in this study should also support the speculation above, indicating that interactions were more specialized than expected based on null models generated according to encounter probability.

The degree of nestedness in the three networks was better explained by partner diversity than by niche overlap. This result indicates that the nestedness degree is more affected by the relative number of specialized interactions between visitors and plants than by the similarity of the plant species subsets used by visitor species. It also suggests that female and male subnetworks have considerably different specialized interactions, synergistically boosting interaction diversity. The greater nestedness of male subnetworks is hidden when seen as a species network. By separating flower–visitor networks into two subnetworks according to visitor sex, we can significantly improve our understanding of flower–visitor networks. Because all males are born from females, their populations are determined by conspecific females rather than floral resources. This more negligible dependence of males on floral resources may affect both temporal and spatial variations in community diversity and pollination services. However, the observed difference in the species composition between female and male subnetworks indicates that results should inevitably include the effects of species and other factors other than sex. Future studies should carry out more careful and deeper analyses and investigate the effects of sexual interactions between male and female visitors on network structure and the significance of sex in trophic and other antagonistic networks.

## CONFLICT OF INTEREST

The author declares no conflict of interest.

## AUTHOR CONTRIBUTION

**Shigeki Kishi:** Conceptualization (lead); Data curation (lead); Formal analysis (lead); Funding acquisition (lead); Investigation (lead); Methodology (lead); Project administration (lead); Resources (lead); Software (lead); Supervision (lead); Validation (lead); Visualization (lead); Writing – original draft (lead); Writing – review & editing (lead).

### OPEN RESEARCH BADGES

This article has earned an Open Data Badge for making publicly available the digitally‐shareable data necessary to reproduce the reported results. The data is available at https://doi.org/10.5061/dryad.hdr7sqvd1.

## Supporting information

Table S1

Table S2

## Data Availability

The datasets are archived at https://doi.org/10.5061/dryad.hdr7sqvd1.
