# Supplementary material for: Nested structure is dependent on visitor sex in the flower‒visitor networks in Kyoto, Japan
Source: Ecol Evol. 2022 Mar 22;12(3):e8743. doi: 10.1002/ece3.8743 (PMC8939293; doi:10.1002/ece3.8743)
Supplement: Supplementary file 1 — Table S1 [file ECE3-12-e8743-s001.docx]

**Supplementary Table S1**

Network size (number of visitor species, number of plant species, total number of species, and total number of interactions (individuals) in each network) and number of modules in each sex-unseparated network (Species), female subnetwork (Female), and male subnetwork (Male).

| Site-Year | Type | No. visitor sp. | No. plant sp. | Total sp. | Total interactions | No. modules |
| --- | --- | --- | --- | --- | --- | --- |
| Ashu1984 | Species | 190 | 48 | 238 | 458 | 19 |
|  | Female | 117 | 40 | 157 | 248 | 13 |
|  | Male | 107 | 34 | 141 | 210 | 18 |
| Ashu1985 | Species | 69 | 29 | 98 | 168 | 7 |
|  | Female | 46 | 26 | 72 | 124 | 7 |
|  | Male | 31 | 15 | 46 | 44 | 10 |
| Ashu1986 | Species | 247 | 45 | 292 | 531 | 18 |
|  | Female | 144 | 41 | 185 | 356 | 14 |
|  | Male | 130 | 32 | 162 | 175 | 15 |
| Ashu1987 | Species | 122 | 25 | 147 | 217 | 13 |
|  | Female | 78 | 22 | 100 | 145 | 14 |
|  | Male | 57 | 16 | 73 | 72 | 12 |
| Kibune1984 | Species | 249 | 56 | 305 | 687 | 26 |
|  | Female | 159 | 51 | 210 | 395 | 24 |
|  | Male | 129 | 44 | 173 | 292 | 30 |
| Kibune1985 | Species | 167 | 47 | 214 | 362 | 23 |
|  | Female | 102 | 42 | 144 | 207 | 17 |
|  | Male | 90 | 32 | 122 | 155 | 13 |
| Kibune1986 | Species | 227 | 44 | 271 | 616 | 24 |
|  | Female | 149 | 40 | 189 | 364 | 15 |
|  | Male | 105 | 30 | 135 | 252 | 15 |
| Kibune1987 | Species | 259 | 76 | 335 | 1098 | 12 |
|  | Female | 168 | 69 | 237 | 740 | 7 |
|  | Male | 147 | 49 | 196 | 358 | 23 |
| KyotoU1985 | Species | 89 | 57 | 146 | 384 | 10 |
|  | Female | 60 | 45 | 105 | 253 | 16 |
|  | Male | 53 | 40 | 93 | 131 | 14 |
| KyotoU1986 | Species | 120 | 51 | 171 | 468 | 22 |
|  | Female | 80 | 46 | 126 | 287 | 14 |
|  | Male | 61 | 31 | 92 | 181 | 10 |
| KyotoU1987 | Species | 71 | 42 | 113 | 223 | 12 |
|  | Female | 44 | 31 | 75 | 137 | 13 |
|  | Male | 44 | 29 | 73 | 86 | 12 |
