# Supplementary material for: Nested structure is dependent on visitor sex in the flower‒visitor networks in Kyoto, Japan
Source: Ecol Evol. 2022 Mar 22;12(3):e8743. doi: 10.1002/ece3.8743 (PMC8939293; doi:10.1002/ece3.8743)
Supplement: Supplementary file 2 — Table S2 [file ECE3-12-e8743-s002.docx]

**Supplementary Table S2**

List of network hub species, defined as by Olesen et al. (2007), in sex-unseparated species networks (Species) and sex-separated subnetworks (Female, female subnetworks; Male, male subnetworks).

| Site | Year | Species | | Female | | Male | |
| --- | --- | --- | --- | --- | --- | --- | --- |
| Ashu | 1984 | Dip | *Melanostoma scalare* | Hym | *Hylaeus globula* | Hemi | *Eurystylus coelestialium* |
|  |  | Dip | *Eristalis cerealis* | Hym | *Bombus diversus diversus* | Dip | *Eristalis cerealis* |
|  |  |  |  |  |  | Hym | *Hylaeus globula* |
|  | 1985 |  | *-* |  | *-* |  | *-* |
|  | 1986 | Hym | *Lasioglossum (Evylaeus) apristum* | Hym | *Lasioglossum (Evylaeus) apristum* |  |  |
|  |  |  |  | Hym | *Andrena hikosana* |  |  |
|  | 1987 |  | *-* |  | *-* | Dip | *Eristalis cerealis* |
| Kibune | 1984 | Hym | *Lasioglossum (Evylaeus) sibiriacum* | Hym | *Vespa simillima xanthoptera* | Hemi | *Onomaus lautus* |
|  |  | Hym | *Hylaeus globula* | Hym | *Bombus diversus diversus* |  |  |
|  |  | Hym | *Vespa simillima xanthoptera* |  |  |  |  |
|  |  | Coleo | *Nonartha cyaneum* |  |  |  |  |
|  |  | Coleo | *Epuraea (Micruria) bergeri* |  |  |  |  |
|  |  | Dip | *Eristalis cerealis* |  |  |  |  |
|  |  | Hemi | *Eurystylus coelestialium* |  |  |  |  |
|  | 1985 |  | *-* | Coleo | *Nonartha cyaneum* |  | *-* |
|  | 1986 | Hym | *Lasioglossum (Hemihalictus) transpositum* | Hym | *Bombus diversus diversus* |  | *-* |
|  |  | Hym | *Lasioglossum (Evylaeus) apristum* | Hym | *Lasioglossum (Evylaeus) apristum* |  |  |
|  |  | Dip | *Episyrphus balteatus* | Hym | *Apis cerana japonica* |  |  |
|  |  |  |  | Dip | *Episyrphus balteatus* |  |  |
|  | 1987 | Hym | *Bombus hypocrita* | Hym | *Vespa simillima xanthoptera* |  | *-* |
|  |  | Hym | *Bombus ardens ardens* | Hym | *Bombus ardens ardens* |  |  |
|  |  | Hym | *Vespa simillima xanthoptera* | Hym | *Apis cerana japonica* |  |  |
|  |  | Dip | *Melanostoma scalare* | Dip | *Episyrphus balteatus* |  |  |
|  |  | Dip | *Cheilosia sp1* |  |  |  |  |
|  |  | Dip | *Cheilosia sp2* |  |  |  |  |
|  |  | Dip | *Tachinidae sp.* |  |  |  |  |
| Kyoto Univ | 1985 |  | *-* |  | *-* |  | *-* |
|  | 1986 | Hym | *Xylocopa appendiculata* | Hym | *Formica japonica* | Coleo | *Cetonia pilifera* |
|  |  | Hym | *Formica japonica* |  |  |  |  |
|  |  | Coleo | *Gametis jucunda* |  |  |  |  |
|  | 1987 |  | *-* |  | *-* |  | *-* |

*Order of each hub species is expressed as either of Coleoptera (Coleo), Diptera (Dip), Hemiptera (Hemi), and Hymenoptera (Hym).
